# Incidence and Mortality Life-Attributable Risks for Patients Subjected to Recurrent CT Examinations and Cumulative Effective Dose Exceeding 100 mSv

**DOI:** 10.3390/clinpract14040125

**Published:** 2024-08-10

**Authors:** Entesar Z. Dalah, Ahmed B. Mohamed, Usama M. Al Bastaki, Sabaa A. Khan

**Affiliations:** 1 HQ Diagnostic Imaging Department, Dubai Health, Dubai, United Arab Emirates; umalbastaki@dubaihealth.ae; 2 College of Medicine, Mohammed Bin Rashid University, Dubai Health, Dubai, United Arab Emirates; 3 Medical Imaging Department, Rashid Hospital, Dubai Health, Dubai, United Arab Emirates; abmohamed@dubaihealth.ae; 4 Medical Imaging Department, Latifa Hospital, Dubai Health, Dubai, United Arab Emirates; saakhan@dubaihealth.ae

**Keywords:** computed tomography (CT), follow-up CT, radiological exams, cumulative effective dose (CED) ≥ 100 mSv, life attributable risk (LAR), recurrent CT

## Abstract

Computed tomography (CT) multi-detector array has been heavily utilized over the past decade. While transforming an individual’s diagnosis, the risk of developing pathogenesis as a result remains a concern. The main aim of this institutional cumulative effective dose (CED) review is to highlight the number of adult individuals with a record of CED ≥ 100 mSv over a time span of 5 years. Further, we aim to roughly estimate both incidence and mortality life-attributable risks (LARs) for the shortlisted individuals. CT studies performed over one year, in one dedicated trauma and emergency facility, were retrospectively retrieved and analyzed. Individuals with historical radiological CED ≥ 100 mSv were short-listed. LARs were defined and established based on organ, age and gender. Out of the 4406 CT studies reviewed, 22 individuals were found with CED ≥ 100 mSv. CED varied amongst the short-listed individuals, with the highest CED registered being 223.0 mSv, for a 57-year-old male, cumulated over an average study interval of 46.3 days. The highest median mortality risk was for females, 214 per 100,000 registered for the age group 51–60 years. While certain clinical indications and diseases require close follow-up using radiological examinations, the benefit-to-risk ratio should be carefully considered, particularly when CT is requested.

## 1. Introduction

The past decade witnessed a global increase in utilizing medical imaging [1]. In particular, the multi-detector array technology of computed tomography (CT) has revolutionized individual diagnosis [2], empowering surgery outcomes [3], improving cancer management and treatment [4], and enhancing the management of stroke cases [5] and cardiac conditions treatments [6]. One major associated concern is the increased population radiation-induced risk as a result of increasing exposure to ionizing radiation [1,2,7]. About 0.7% of the cases diagnosed with cancer in 2015 in France were attributable to medical imaging exposure including CT, general X-ray, interventional radiology, and internal nuclear medicine [8]. Out of the 105,802 CT scan cases acquired in Spain in 2013, 168.6 were estimated to encounter cancer during their lifespan due to CT imaging dose [9]. An effective dose of 10 mSv per scan was assumed to result in 1 to every 2000 individuals who would encounter life-attributable risk (LAR) of cancer [10]. A cumulative effective dose (CED) of 100 mSv increases the fatal encountered risk in 1 to every 200 adults [11,12]. Emerging convincing evidence suggests an excess of cancer risk at an organ-effective dose of <100 mSv [13]. Typically, every additional scan increases the chance of cancer risk significantly [14], especially if the frequent acquired exams are with CT [2,15]. To date, there remains a lack of characterized information on individuals with CED from repeated CT exams [16,17]. The main aim of our institutional review is to highlight the number of adult individuals with a record of CED ≥ 100 mSv over a time span of 5 years. Further, an estimate of both incidence and mortality LARs for the shortlisted individuals will be demonstrated.

## 2. Materials and Methods

This retrospective study was approved by our Institutional Scientific Research Ethics Committee. Data collection started by initially retrieving information over 12 month period for the year 2021. Information collected included CT studies, individual CED, average days interval between radiological studies, individual radiological history, and individual age and gender.

### 2.1. Short-Listing Individuals with CED ≥ 100 mSv

**Figure 1:**
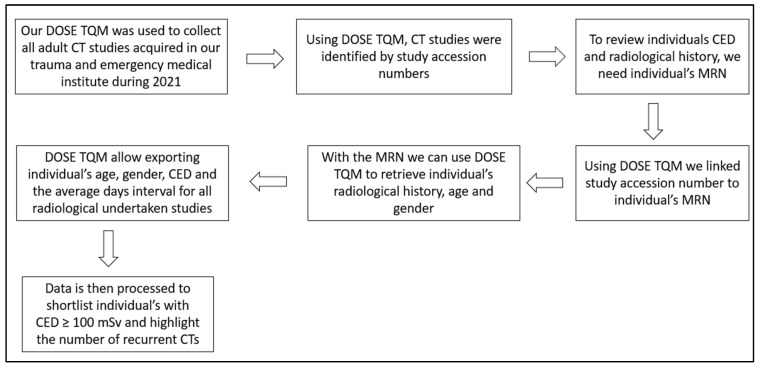
Data retrieval chart.

The data mentioned above were generated using our institute’s electronic radiation dose monitoring and archiving system, DOSE TQM (Qaelum NV, Belgium) [18]. The DOSE TQM system is linked with our regional healthcare sector’s existing radiology picture archiving and communication system (PACS). DOSE TQM supports different data exporting formats. However, the exported data are identified by study (e.g., study accession number, study identifier (ID) or study unique identifier (UID)), not by individual name or individual medical registration number (MRN). This is in place to maintain individual privacy. Furthermore, neither DOSE TQM nor PACS supports automated retrieval of CED. Hence, study accession numbers were used to identify individual MRNs. Once all the CT studies were linked with individual MRN, DOSE TQM was used to retrieve individual radiological history. This allowed us to access and analyze individuals’ CED and the average day’s interval for all the radiological studies undertaken by each individual. All data exported from DOSE TQM were in Excel format. Patient medical history information was retrieved from our radiology information system. Figure 1 demonstrates the steps undertaken to short-list adult individuals with CED ≥ 100 mSv. Initially, data retrieval was based on the CT studies performed at our dedicated trauma and emergency medical institute. However, retrieving individual radiological history demanded the inclusion of all radiological services performed at any medical institute within our healthcare sector.

### 2.2. Inclusion Criteria

Short-listing inclusion criteria were limited to adults (male and female) that underwent ionizing radiation radiological exams, apart from nuclear medicine, with a CED dose of 100 mSv and above that were averaged over a 5-year period.

### 2.3. Organ Dose Estimate

To estimate the dose of targeted organs, the commercial software VirtualDose^TM^ CT [19] was used. VirtualDose^TM^ CT is a Monte Carlo-based computational method that is designed to model various CT scanners including X-ray energy spectrum, beam width and collimation, filtration and tube current, and clinical protocols. VirtualDose^TM^ CT supports a comprehensive range of organ dose and effective dose algorithms that are recommended in the International Commission Radiation Protection Report 60 [20] as well as in the more recent ICRP report 103 [21]. VirtualDose^TM^ CT provides organ dose estimates with up to 14% proposed uncertainty [22].

### 2.4. Assessment of Lifetime Attributable Risk Incidence and Mortality

A rough LAR of cancer incidence and mortality was estimated for individuals with CED ≥ 100 mSv using tables 12D-1 and 12D-2, respectively, provided in [11].

### 2.5. Statistical Analysis

The statistical analysis was performed using GraphPad Prism v8.0 Software (La Jolla, CA, USA). The number of undertaken radiological exams for the short-listed individuals with CED ≥ 100 mSv was presented in the median. The dose of the targeted organs was presented in median, standard deviation (SD) and interquartile range (IQR, 25th–75th percentile) for both genders based on age groups. The cumulative dose of targeted organs was presented in median and IQR based on age and gender. Estimates of LAR of cancer incidence and mortality were demonstrated per 100,000 for CT-associated exams only.

## 3. Results

A total of 4406 adult CT studies were performed between the period of 1 January 2021 to 31 December 2021 in our dedicated trauma and emergency hospital. The CT exams performed (and number of cases, %) included: abdomen and pelvis + contrast (1438, 32.6%), polytrauma (1038, 23.6%), kidney ureter bladder (KUB) (723, 16.4%), brain stroke with CT angiogram (663, 15.0%), chest abdomen and pelvis + contrast (357, 8.1%), triphasic liver (99, 2.2%), and abdomen angiogram (88, 2.1%). Table 1 summarizes all CT studies in relation to age, gender and the number of recurrent CT exams per study.

### 3.1. Individuals with CED ≥ 100 mSv

The adult CT exam recurrent rate in 2021 was found to be ~5.6% (248 recurrent CTs out of the total 4406 adult CTs). In total, 22 individuals were found with CED ≥ 100 mSv. Looking into the radiological history of the 22 short-listed individuals, the CED was mainly attributed to recurrent CT studies with minimal contribution from general/interventional radiology. Table 2 demonstrates a summary of patient medical history, CED in mSv, average days interval between the undertaken studies, and the total number of CT and conventional/interventional radiology studies found for every short-listed individual.

The 22 short-listed individuals with CED ≥ 100 mSv were divided into 8 age groups to facilitate the use of tables 12D-1 and 12D-2, respectively, provided in [11]. Groups were organized as 15–20, 21–30, 31–40, 41–50, 51–60, 61–70, 71–80, and 81–90-years-old. Table 3 presents a descriptive summary of the CED in mSv, average days interval between the undertaken studies, and gender distribution per age group.

The age range of the 22 short-listed individuals with CED ≥ 100 mSv was 22 to 81 years old. The highest number of recurrent CT was demonstrated for the age group 31 to 40 years old, and the lowest for the age group of 81 to 90. About 14% of the short-listed individuals are in the age group 21 to 30 years old.

CED varied amongst the short-listed individuals, with the highest CED registered being 223.0 mSv, for a 57-year-old male, cumulated over an average study interval of 46.3 days. This individual suffered from a liver cholangiocarcinoma, the individual underwent 3 general X-ray procedures and 4 triphasic liver CT exams. The lowest CED registered amongst the 22 short-listed individuals, was ~102 mSv cumulated over an average study interval of 25.2 days. The 76-year-old male suffered from colon cancer, pulmonary embolism and retroperitoneal hematoma. The individual was subjected to 11 CT exams and 23 general X-ray procedures. On the other hand, we registered a CED of 214.8 mSv for a 26-year-old male that cumulated over an average study interval of 1.65 days. Out of the 22 short-listed individuals, 5 were female patients and 17 were males as shown in Table 2. Table 3 illustrates a descriptive summary of the CED per age group, average day intervals and gender.

### 3.2. Dose of Targeted Organs

Table 4 demonstrates a descriptive summary of targeted organ doses. Where the median, median, and IQR of the cumulative dose values for nine targeted organs have been calculated using tissue weighting factors reported in [20]. Generally, the cumulative median and median dose for all targeted organs in all age groups were below 100 mGy, except for a few cases. A 59-year-old female who underwent three enhanced abdomen and pelvis CT exams registered a median cumulative dose > 100 mGy in the following organs: bladder, colon, liver, stomach and uterus. The 75th percentile cumulative dose of liver, lung and stomach for the male group 61–70-year-old were observed to be > 100 mGy.

### 3.3. Short-Listed LAR of Incidence and Mortality

Table 5 shows the estimated LAR of incidence per 100,000 patients that resulted from the recurrent CT exams categorized based on age, gender and target organs. An example is estimating the LAR incidence of, e.g., lung cancer. Following lung organ dose estimation and calculating the cumulative lung dose, age and gender-specific LAR coefficient provided in Table 12D-1 in the BEIR VII Phase 2 [11] were used to estimate lung cancer incidence. Linear interpolation was used to roughly estimate the LAR of incidence coefficient for the ages not prescribed in Table 12D-1 in [11]. Amongst the 22 short-listed individuals, the lowest LAR incidence was associated with the thyroid gland for both genders at all age groups.

Table 6 summarizes age and gender-specific estimated life-attributable cancer incidence and mortality per 100,000 patients that resulted from the recurrent CT exams. Table 6 reports the LAR of incidence using the cumulative dose of all organs listed in Table 5 (males and females). The highest median mortality risk of 1.24% (i.e., 124 per 100,000) of patients registered for males was for the age group 61–70 years. For females, the highest median mortality risk of 2.14% (i.e., 214 per 100,000) patients was recorded in the age group 51–60 years.

## 4. Discussion

In this study, the CT recurrent rate was found to be ~5.6% (248 recurrent CTs out of the total 4406 adult CTs). Only 22 patients were found with CED ≥ 100 mSv that resulted from recurrent CT exams cumulated over a time span of 5 years.

The median CED varied considerably between the seven age groups for both males and females as shown in Table 4. The highest median CED for males was 185.75 mSv recorded for the age group 21–30 years. For females, the highest median CED was 190.10 mSv registered for the age group 61–70 years. In contrast, the lowest median CED for males was 119.20 mSv registered for the age group 31–40 years. For females, the lowest median CED was 103.30 mSv registered for the age group 51–60 years.

Clinically, it is not uncommon to witness individuals with CED records exceeding 100 mSv. This is particularly known for certain clinical conditions. Examples of this are hemodialysis patients and patients undergoing endovascular aortic repair. De Mauri et al. [23] reported that about 16% of their hemodialysis cohort patients registered CED > 100 mSv after 3 years of follow-up. Coyle et al. [24] reported that 23% of their hemodialysis cohort patients registered CED > 100 mSv over 4 years of follow-up. Brambilla et al. [25] reported an annual CED of 104 mSv in about 93% of the patients who underwent endovascular repair of aortic aneurysms and were subjected to recurrent cardiac angiogram CT follow-ups. Kalender et al. [26] reported a maximum CED of 310 mSv, given that each patient on average underwent four cardiac angiogram CT exams. Zewde and team [27] reported a general overview of patients subjected to recurrent CT exams, showing a wide range of the received CED from 100–1185 mSv. Evidently, certain clinical conditions call for a repeated radiological examination and follow-up. In the present study, a 22-year-old male who suffered from necrotizing pancreatitis underwent a total of 33 radiological exams (5 CTs and 28 general X-rays) at a study average interval of 1.2 days, registering a total CED of 156.7 mSv with 98.8% of the total CED being attributable to the five CT exams.

On a similar note, the cumulative dose calculated for the 16 targeted organs also varied between the seven age groups, Table 3. The highest median bladder cumulative dose recorded among our 22 cohort patients was 68.43 mGy registered for the age group 41–50 years. The highest median colon cumulative dose recorded in our sample was 75.37 mGy registered for the age group 41–50 years. Similarly, the highest median liver, stomach and kidneys cumulative dose recorded were 77.01, 50.91 and 79.40 mGy, respectively, all registered for the age group 41–50 years. Examples of organs registering high 75th percentile cumulative dose (i.e., >100 mGy) in our study include: the liver (183.10 mGy), kidneys (181.72 mGy), colon (172.12 mGy), bladder (147.32 mGy) and stomach (126.32 mGy). All of the organs listed above with high 75th percentile organ cumulative dose were registered for the age group 51–60 years. Organs with high-end cumulative doses existed in the literature. Brambilla et al. [25] reported median cumulative organ doses ranging from 191 to 271 mGy for the lung, bone marrow, liver, colon and stomach. Zewde et al. [27] reported median cumulative organ doses ranging from 121.7 to 220.0 mGy for the bone marrow, esophagus, colon, lung, liver and stomach. Certainly, the higher the total cumulative dose, the higher the organ’s cumulative dose. Further, organ cumulative doses are subjected to the patient clinical condition and indications which determine the choice of the acquired exam protocol. This is particularly relevant in CT exams, with CT dose indices showing to vary between the different clinical indications acquired using CT head, CT abdomen and pelvis and CT KUB [28].

The median and IQR LAR incidence varied across the nine organs, and is reported in Table 5. The highest median LAR of incidence registered was for colon cancer ~0.9% (90 per 100,000 male patients) between the age group 41–50 years. For females, the highest median LAR of incidence was also for colon cancer 1.41% (141 per 100,000 female patients) between the age group 51–60 years. The highest median LAR based on all nine organs listed in Table 5 was registered for the female group aged 51–60 years, 4.04% (404 per 100,000 patients). For males, the highest median LAR based on all the nine organs was registered for the age group 41–50 years, 2.07% (207 per 100,000) patients. Dalah et al. [29] reported (0.059%, 5.89 per 100,000 patients) the LAR incidence of lung cancer in female patients subjected to CT chest exam in their 60s. Further, the risk of inducing lung cancer as a result of undergoing sequential CT cardiac angiogram and calcium-scoring CT exam was found to be the greatest for females in their 50s [30].

In the present study, the highest median mortality risk of 1.24% (i.e., 124 per 100,000) patients for males was observed between the age group 61–70 years. This was followed by 90 per 100,000 patients between the age group 41–50 years and 61 per 100,000 patients between the age group 21–30 years. The lowest median LAR of mortality registered for males was 20 per 100,000 between the age group 31–40 years. For females, the highest median mortality risk of 2.14% (i.e., 214 per 100,000) patients was recorded between age group 51–60 years. This was followed by 112 per 100,000 patients witnessed between the age group 71–80 years and 54 per 100,000 patients observed between the age group 61–70 years. The lowest median LAR of mortality recorded for females was 24 per 100,000 between the age group 21–30 years. In line with this, Zewde et al. [27] report the highest median LAR of death 0.7 (in males) and 0.8 (in females) per 100,000 patients in their 20s.

This is the first CED and recurrent CT review investigation in our institute. To the authors’ knowledge, limited studies reported individual-based categorized CED from recurrent CT exams. In addition, we presented an overview of LARs associated with the CED from recurrent CT over a time span of 5 years. The CED and organ dose levels reported in this review draw attention to the increased cancer incidence and mortality in our population.

Radiation dose optimization is an objective every single radiology department strives to achieve. An effective clinical dose optimization demands a well-structured dose-level system, one that is based on dose per scan acquisition for every CT protocol [31]. Such a dose-structured system allows better control and reduces the impact of confounding factors. Further, implementing referral guidelines and a well-structured justification system in day-to-day practice is of great importance in controlling the number of recurrent CTs for individuals [28].

This study encountered several limitations. Because of the fact that the initial retrieval of data was limited to one medical center, knowledge on regional and national cumulative dose remains uncertain, likewise insights on CT recurrent information. The inclusion criteria were limited to adult patients with CED ≥ 100 mSv. In fact, pediatric and young adults should receive the utmost attention. The CED data reported here might not reflect the actual total CED received by an individual, particularly if an individual had received a radiological service in a private medical center. This is because private PACS systems are not linked with our governmental health sector PACS system. The present study demonstrated an estimate of LAR incidence and mortality rate on an individual-specific basis that resulted from recurrent CT exams only. While this could result in an underestimation of LAR incidence and mortality rate, the contribution of general radiology to the total CED per individual is usually below 1% [25]. Variables exist in estimating organ doses [32], and this is a vital source of uncertainty especially since organ dose is used to estimate the incidence and mortality of LARs. Not only the tool used to estimate organ doses is a source of variability, the scan range is also a contributing factor. Determining patients with CED ≥ 100 mSv over a large scale remains a time-consuming task and is technically challenging. The dose monitoring tool allows the filtering and retrieval of the effective doses but not CEDs.

## 5. Conclusions

The CED and organ dose levels reported in this review draw attention to the risk of increased cancer incidence and mortality in our population. Certain clinical indications and diseases require close follow-up using radiological examinations. This pegs the establishment of a scan acquisition dose system for every single CT protocol, and the implementation of systematic justification and guidelines. Higher CED are directly associated with increasing the LAR incidence and mortality rate as a result of increasing organ doses.

## Figures and Tables

**Table 1:** CT study findings and patient characteristics for the chosen sample.

CT Study/Number of CT Studies = 4406	Gender	Age (Years)	Number of Recurrent CT
	(No. Male and No. Female)	Median ± SD (Range)	
Abdomen/Pelvis with contrast, 1438	771 M, 667 F	48.2 ± 18.3 (22–80)	104
Polytrauma, 1038	932 M, 106 F	28 ± 8.5 (22–34)	34
KUB, 723	490 M, 233 F	59 ± 18.7 (39–76)	3
Brain Stroke with CTA, 663	486 M, 177 F	63.8 ± 13.9 (40–80)	18
Chest/Abdomen/Pelvis with contrast, 357	203 M, 154 F	68.5 ± 7.9 (55–80)	40
Triphasic Liver, 99	54 M, 45 F	56.2 ± 13.4 (34–70)	29
Abdominal Angiogram, 88	55 M, 33 F	44.3 ± 22.7 (22–75)	20

KUB: Kidney–Ureter–Bladder; CTA: Computed Tomography Angiography.

**Table 2:** Summary of individuals with CED ≥ 100 mSv including radiological history, average days interval, patient age and gender.

#	Gender/Age Range (Years)	History	Total Cumulative Effective Dose (mSv)	Average Study Interval (Days)	Radiological Exams		
					Total	CTs	General X-ray
1	M/46	Post right hemicolectomy and ileocolic anastomosis for perforated colon complicated with duodenal injury	231.7	3.0	32	11	21
2	M/57	Liver tumor (cholangiocarcinoma	223.0	46.3	7	4	3
3	M/71–73	Metastatic colon cancer with liver metastases post multiple surgeries	220.9	16.2	36	9	27
4	M/25–26	Necrotizing pancreatitis	214.8	1.7	61	7	53
5	F/69–70	Admitted with perianal abscess. Hospital course was complicated post-surgical by sepsis, intraabdominal collections	190.1	8.4	35	12	23
6	M/56–57	Embolic superior mesenteric artery with bowel ischemia, Complicated exploratory laparotomy followed by multiple abdominal surgeries	164.9	1.7	22	6	16
7	M/22	Necrotizing pancreatitis	156.7	1.2	33	5	28
8	M/33–34	Necrotizing pancreatitis	149.9	4.2	19	8	11
9	M/62–63	Decompensated liver cirrhosis complicated by metastatic HCC and Upper GI bleeding	142.9	11.6	21	6	15
10	M/62–63	Pancreas cancer	132	36.9	10	7	3
11	M/43–46	Bariatric surgery/sleeve gastrectomy, complicated by leakage and abscess formation	129.4	38	17	3	14
12	F/22–23	Polytrauma with pelvic and femur fractures	122.5	3.0	16	3	13
13	M/79–81	Cholangiocarcinoma, right lung adenocarcinoma post lobectomy and renal leiomyosarcoma	121.9	41.3	23	7	16
14	M/34	Polytrauma with pelvic bone fractures and active pelvic bleeding	121.1	2.9	8	3	5
15	M/38–40	Decompensated liver cirrhosis complicated by bacterial peritonitis	117.3	19.9	28	8	20
16	M/61	Decompensated liver cirrhosis with HCC	112.3	25	4	2	2
17	F/70–72	Breast cancer	112.3	107.2	9	3	6
18	F/33–34	Class 3 severe obesity + DM + HTN and infertility	108.1	195.3	4	2	2
19	M/54–55	Lumbar Spondylodiskitis + mediastinal lymphadenopathy	103.3	12.9	20	5	15
20	F/58–59	DM + HTN + NAFLD/NASH + small colonic polyp and internal hemorrhoids (by colonoscopy) + adrenal adenoma	103.3	200	3	2	1
21	M/37–39	Abdominal wall hernia, surgery complicated by abdominal wall collection	102.9	120.8	9	4	5
22	M/73–76	Operated colon cancer + pulmonary embolism + retroperitoneal hematoma + DM and HTN	101.9	25.2	35	12	23

HCC: Hepatocellular carcinoma; DM: Diabetes Mellitus; HTN: Hypertension; NAFLD: Nonalcoholic Fatty Liver; NASH: Nonalcoholic Steatohepatitis. Note: the CED registered for individual number 4 included one interventional radiology procedure.

**Table 3:** Median and IQR dose, based on CT exams only, for 16 organs classified per age group.

No.	Organs	Age Groups (Years), Number of Cases Out of 100%						
		21–30, 14%	31–40, 23%	41–50, 9%	51–60, 18%	61–70, 18%	71–80, 14%	81–90, 5%
		Chest, Abdomen, Pelvis	Chest, Abdomen, Pelvis, Brain, Whole-Body	Chest, Abdomen, Pelvis, Brain	Chest, Abdomen, Pelvis, Brain	Chest, Abdomen, Pelvis, Brain	Chest, Abdomen, Pelvis, Brain	Chest, Abdomen, Pelvis, Brain
		Organ Dose in Median (mGy) IQR (25th–75th percentile)						
1	Bladder	31.38 (21.74–31.45)	19.31 (5.16–55.84)	68.43 (51.12–85.74)	15.13 (5.21–147.32)	20.83 (21.73–40.01)	1.25 (18.2–31.7)	5.60 (0.05–11.74)
2	Bone-marrow (red)	9.38 (8.44–15.48)	7.53 (2.97–35.07)	27.46 (21.24–33.67)	10.48 (5.34–56.45)	8.49 (10.68–43.11)	2.34 (21.01–28.86)	2.59 (0.22–5.14)
3	Brain	0.22 (0.15–0.51)	0.23 (0.15–95.06)	5.07 (3.06–7.07)	7.20 (1.01–15.62)	0.23 (0.85–21.89)	0.99 (1.75–132.47)	0.12 (0.01–1.91)
4	Breast (female)	1.61 (0.81–5.51)	1.54 (1.14–6.61)	6.19 (5.42–6.95)	9.46 (2.48–21.70)	1.77 (6.16–92.64)	0.34 (2.14–57.58)	0.49 (0.05–1.08)
5	Colon	23.59 (16.91–52.98)	21.09 (6.53–75.50)	75.37 (56.33–94.42)	19.62 (8.55–172.12)	25.32 (28.31–81.47)	2.53 (19.93–56.23)	6.25 (0.01–15.92)
6	Gonads	29.65 (21.93–37.43)	17.75 (6.03–49.10)	55.71 (46.86–64.55)	13.00 (1.19–141.99)	15.81 (12.24–21.84)	1.44 (21.59–33.9)	4.72 (0.01–10.80)
7	Liver	24.03 (5.59–66.18)	21.54 (8.02–79.18)	77.01 (57.71–96.33)	28.26 (10.64–183.10)	27.30 (32.42–125.77)	3.38 (21.09–72.92)	6.35 (0.06–16.74)
8	Lung	2.96 (1.02–9.13)	3.21 (2.03–11.75)	10.51 (8.65–12.36)	15.52 (3.50–28.11)	3.32 (8.14–95.60)	0.58 (3.65–62.61)	0.82 (0.10–2.03)
9	Esophagus	0.59 (0.3–1.97)	0.84 (0.41–23.95)	3.82 (3.32–4.32)	6.09 (2.93–8.58)	0.66 (3.94–32.94)	0.94 (12.01–26.31)	0.32 (0.05–0.93)
10	Salivary glands	0.39 (0.25–0.92)	0.36 (0.26–83.70)	6.64 (4.23–9.06)	5.05 (1.81–17.10)	0.41 (1.67–20.75)	2.20 (3.44–104.26)	0.20 (0.01–2.51)
11	Skin	9.35 (8.88–16.36)	7.29 (2.88–33.34)	27.50 (38.35–63.48)	9.87 (4.69–54.26)	8.52 (10.21–38.42)	2.49 (18.13–26.39)	2.49 (0.24–5.30)
12	Stomach	15.77 (3.87–43.05)	14.19 (5.83–52.29)	50.91 (38.35–63.48)	21.86 (7.88–126.32)	17.92 (22.55–103.25)	2.84 (13.77–63.88)	4.18 (0.07–11.00)
13	Thyroid	0.46 (0.28–1.28)	0.53 (0.32–43.95)	3.64 (2.91–4.36)	3.88 (1.82–4.49)	0.5 (2.98–13.22)	0.46 (9.87–11.69)	0.24 (0.02–0.75)
14	Kidneys	24.76 (5.46–68.45)	22.22 (8.11–81.22)	79.40 (59.44–99.35)	29.20 (10.77–181.72)	28.23 (32.69–126.20)	3.37 (21.76–74.40)	6.55 (0.05–17.28)
15	Lenses of the eye	0.73 (0.48–0.99)	0.85 (0.59–64.79)	4.89 (3.71–6.06)	8.44 (2.38–11.57)	0.78 (1.10–15.49)	0.69 (1.35–87.93)	0.37 (0.02–1.65)
16	Uterus/Cervix (female) or Prostate (male)	34.85 (24.26–35.83)	18.75 (5.73–53.16)	75.66 (56.30–95.01)	15.16 (4.33–128.93)	24.08 (21.46–32.28)	1.76 (20.95–41.73)	5.23 (0.09–12.70)

**Table 4:** Cumulative radiation dose for the organs used to estimate LAR incidence based on age groups and gender distribution.

Age Group (Year) Total Cases = 22	Cumulative Effective Dose (mSv)		Organ Dose (mGy) Median (25th–75th percentile)								
	Median	(Min–Max)	Bladder	Breast	Colon	Liver	Lung	Stomach	Thyroid	Uterus	Prostate
Male (17 cases, 77%)											
21–30, 12%	185.75	156.70–214.80	26.60, (24.17–29.02)	-	38.29 (30.94–45.63)	45.11(34.57–55.64)	6.05(4.50–7.59)	29.41(22.59–36.23)	0.8 (0.67–1.08)	-	29.56 (26.91–32.20)
31–40, 24%	119.20	102.90–149.90	30.05 (7.57–37.13)	-	15.93 (8.64–47.35)	16.24 (9.48–49.36)	3.37 (2.37–7.89)	10.69 (6.51–32.40)	1.14 (0.37–22.90)	-	16.34 (8.45–37.34)
41–50, 12%	180.55	129.40–231.70	68.43 (51.12–85.74)	-	75.38 (56.33–94.42)	77.02 (57.71–96.33)	10.51 (8.65–12.36)	50.91 (38.35–63.48)	3.64 (2.91–4.36)	-	75.66 (56.30–95.01)
51–60, 18%	164.90	103.30–223.00	10.74 (3.36–19.51)	-	18.19 (5.33–21.05)	21.45 (7.03–35.06)	5.37 (2.87–25.67)	14.12 (5.80–29.60)	3.48 (1.26–4.28)	-	8.45 (2.96–21.87)
61–70, 18%	132.00	112.30–142.90	36.87 (20.83–41.06)	-	68.80 (25.32–85.69)	98.88 (27.30–134.73)	73.55 (3.32–102.95)	81.39 (17.92–110.54)	11.46 (0.50–13.80)	-	22.21 (21.21–34.39)
71–80, 12%	161.40	101.90–220.90	24.95 (21.58–28.33)	-	33.83 (26.88–40.77)	47.01 (34.05–59.96)	24.36 (14.00–37.41)	35.48 (24.63–46.34)	11.44 (11.32–11.57)	-	25.10 (23.02–27.17)
81–90, 6%	121.90 ^f^	-	36.43 ^f^	-	49.99 ^f^	52.73 ^f^	6.65 ^f^	34.69 ^f^	2.57 ^f^	-	36.94 ^f^
**Female (5 Cases, 23%)**											
21–30, 20%	122.50	-	31.38 ^a^	0.81 ^a^	16.91 ^a^	5.59 ^a^	1.02 ^a^	3.87 ^a^	0.28 ^a^	35.83 ^a^	-
31–40, 20%	108.10 ^b^	-	21.1 ^b^	1.54 ^b^	24.87 ^b^	25.54 ^b^	3.21 ^b^	17.57 ^b^	0.53 ^b^	18.75 ^b^	-
41–50, 0%	NA	NA	NA	NA	NA	NA	NA	NA	NA	NA	-
51–60, 20%	103.30 ^c^	-	189.2 ^c^	13.72 ^c^	222.48 ^c^	232.44 ^c^	28.92 ^c^	158.56 ^c^	4.56 ^c^	164.62 ^c^	-
61–70, 20%	190.10 ^d^	-	24.44 ^d^	19.31 ^d^	37.26 ^d^	47.78 ^d^	22.61 ^d^	36.41 ^d^	10.42 ^d^	25.95 ^d^	-
71–80, 20%	112.30 ^e^	-	26.39 ^e^	57.58 ^e^	56.23 ^e^	72.89 ^e^	62.61 ^e^	63.88 ^e^	9.87 ^e^	41.73 ^e^	-
81–90, 0%	NA	NA	NA	NA	NA	NA	NA	NA	NA	NA	-

^a^ Median cumulative for a single female patient who underwent 3 CT exams including CT polytrauma-whole-body, enhanced CT abdomen and pelvis, and CT pelvis; ^b^ Median cumulative for a single female patient who underwent 2 CT exams (triphasic liver and enhanced abdomen and pelvis); ^c^ Median cumulative for a single female patient that underwent 3 enhanced abdomen and pelvis CT exams; ^d^ Median cumulative for female that was subjected to 2 CT brains, 2 chest–pelvis derange, and triphasic CT; ^e^ Median cumulative for a single female patient that underwent 2 enhanced chest, abdomen and pelvis CT exams; ^f^ Median cumulative for a single male patient that underwent 3 enhanced abdomen and pelvis CT, triphasic CT and brain CT; NA Not available.

**Table 5:** Life-attributable risk (LAR) incidence based on CT only per gender and age group per 100 patients.

Age Group (Year) Total Cases = 22	%LAR of Incidence per 100 Patients Median (IQR, 25th–75th Percentile)								
	Bladder	Breast	Colon	Liver	Lung	Stomach	Thyroid	Uterus	Prostate
Male (17 Cases, 77%)									
21–30, 12%	0.26 (0.23–0.29)	-	0.60 (0.47–0.74)	0.12 (0.09–0.16)	0.08 (0.06–0.11)	0.11 (0.08–0.13)	0.00 (0.00–0.00)	-	0.13~(0.11–0.140)
31–40, 24%	0.12 (0.06–0.29)	-	0.20 (0.11–0.58)	0.03 (0.02–0.04)	0.04 (0.02–0.08)	0.03 (0.02–0.09)	0.01 (0.00–0.01)	-	0.10~(0.03–0.13)
41–50, 12%	0.53 (0.40–0.66)	-	0.88 (0.66–1.11)	0.15 (0.11–0.19)	0.11 (0.09–0.13)	0.13 (0.10–0.16)	0.00 (0.00–0.00)	-	0.26~(0.20–0.33)
51–60, 18%	0.07 (0.02–0.14)	-	0.04 (0.01–0.05)	0.03 (0.01–0.05)	0.05 (0.03–0.24)	0.03 (0.01–0.07)	0.00 (0.00–0.00)	-	0.02~(0.01–0.06)
61–70, 18%	0.20 (0.13–0.25)	-	0.41 (0.23–0.61)	0.13 (0.04–0.16)	0.62 (0.03–0.83)	0.15 (0.03–0.20)	0.00 (0.00–0.00)	-	0.05~(0.05–0.08)
71–80, 12%	0.10 (0.08–0.12)	-	0.19 (0.14–0.23)	0.03 (0.02–0.04)	0.11 (0.07–0.16)	0.04 (0.03–0.06)	0.00 (0.00–0.00)	-	0.03 (0.02–0.03)
81–90, 6%	0.08 ^f^	-	0.15 ^f^	0.02 ^f^	0.02 ^f^	0.00 ^f^	0.00 ^f^	-	0.02 ^f^
**Female (5 Cases, 23%)**									
21–30, 20%	0.32 ^a^	0.03 ^a^	0.18 ^a^	0.01 ^a^	0.03 ^a^	0.02 ^a^	0.00 ^a^	0.09 ^a^	-
31–40, 20%	0.17 ^b^	0.03 ^b^	0.20 ^b^	0.03 ^b^	0.08 ^b^	0.06 ^b^	0.00 ^b^	0.03 ^b^	-
41–50, 0%	NA	NA	NA	NA	NA	NA	NA	NA	NA
51–60, 20%	1.24 ^c^	0.05 ^c^	1.41 ^c^	0.17 ^c^	0.59 ^c^	0.44 ^c^	0.00 ^c^	0.16 ^c^	-
61–70, 20%	0.11 ^d^	0.02 ^d^	0.17 ^d^	0.02 ^d^	0.33 ^d^	0.07 ^d^	0.00 ^d^	0.01 ^d^	-
71–80, 20%	0.11 ^e^	0.09 ^e^	0.22 ^e^	0.03 ^e^	0.79 ^e^	0.11 ^e^	0.00 ^e^	0.02 ^e^	-
81–90, 0%	NA	NA	NA	NA	NA	NA	NA	NA	NA

^a^ Median cumulative for a single female patient who underwent 3 CT exams including CT polytrauma-whole-body, enhanced CT abdomen and pelvis, and CT pelvis; ^b^ Median cumulative for a single female patient who underwent 2 CT exams (triphasic liver and enhanced abdomen and pelvis); ^c^ Median cumulative for a single female patient that underwent 3 enhanced abdomen and pelvis CT exams; ^d^ Median cumulative for female that was subjected to 2 CT brains, 2 chest-pelvis derange, and triphasic CT; ^e^ Median cumulative for a single female patient that underwent 2 enhanced chest, abdomen and pelvis CT exams; ^f^ Median cumulative for a single male patient that underwent 3 enhanced abdomen and pelvis CT, triphasic CT and brain CT; NA Not available.

**Table 6:** Age group distribution and LAR of Incidence and mortality per 100 patients based on gender.

Age Group (Year) Total Cases = 22	%LAR of Incidence per 100 Patients		%LAR of Mortality per 100 Patients	
	Range (Min–Max)	Median	Range (Min–Max)	Median
Male (17 Cases, 77%)				
21–30, 12%	0.79–1.83	1.31	0.33–0.88	0.61
31–40, 24%	0.10–3.05	0.46	0.07–1.52	0.20
41–50, 12%	1.04–3.09	2.07	0.46–1.34	0.90
51–60, 18%	0.12–0.50	0.34	0.10–0.44	0.23
61–70, 18%	0.52–1.92	1.79	0.25–1.60	1.24
71–80, 12%	0.22–0.78	0.50	0.13–0.59	0.36
81–90, 6%	-	0.30 ^f^	-	0.30 ^f^
**Female (5 Cases, 23%)**				
21–30, 20%	-	0.69 ^a^	-	0.24 ^a^
31–40, 20%	-	0.60 ^b^	-	0.28 ^b^
41–50, 0%	NA	NA	NA	NA
51–60, 20%	-	4.04 ^c^	-	2.14 ^c^
61–70, 20%	-	0.74 ^d^	-	0.54 ^d^
71–80, 20%	-	1.36 ^e^	-	1.12 ^e^
81–90, 0%	NA	NA	NA	NA

^a^ Median cumulative for a single female patient who underwent 3 CT exams including CT polytrauma-whole-body, enhanced CT abdomen and pelvis, and CT pelvis; ^b^ Median cumulative for a single female patient who underwent 2 CT exams (triphasic liver and enhanced abdomen and pelvis); ^c^ Median cumulative for a single female patient that underwent 3 enhanced abdomen and pelvis CT exams; ^d^ Median cumulative for female that was subjected to 2 CT brains, 2 chest–pelvis derange, and triphasic CT; ^e^ Median cumulative for a single female patient that underwent 2 enhanced chest, abdomen and pelvis CT exams; ^f^ Median cumulative for a single male patient that underwent 3 enhanced abdomen and pelvis CT, triphasic CT and brain CT; NA Not available.

## Data Availability

All the necessary data has been presented here.
